# Potential for *Methanosarcina* to Contribute to Uranium Reduction during Acetate-Promoted Groundwater Bioremediation

**DOI:** 10.1007/s00248-018-1165-5

**Published:** 2018-03-02

**Authors:** Dawn E Holmes, Roberto Orelana, Ludovic Giloteaux, Li-Ying Wang, Pravin Shrestha, Kenneth Williams, Derek R Lovley, Amelia-Elena Rotaru

**Affiliations:** 1 Department of Physical and Biological Science, Western New England University, Springfield, MA USA; 2 Department of Microbiology, University of Massachusetts Amherst, Amherst, MA USA; 3 Department of Molecular Biology and Genetics, College of Agriculture and Life Sciences, Cornell University, Ithaca, NY USA; 4 Energy Biosciences Institute, University of California Berkeley, Berkeley, CA USA; 5 Lawrence Berkeley National Laboratory, Berkeley, CA USA; 6 University of Southern Denmark, Campusvej 55, 5240 Odense, Denmark

**Keywords:** Uranium bioremediation, Methanogenesis, U(VI) reduction, Acetate amendment, *Methanosarcina*

## Abstract

Previous studies of acetate-promoted bioremediation of uranium-contaminated aquifers focused on *Geobacter* because no other microorganisms that can couple the oxidation of acetate with U(VI) reduction had been detected in situ. Monitoring the levels of methyl CoM reductase subunit A (*mcrA*) transcripts during an acetate-injection field experiment demonstrated that acetoclastic methanogens from the genus *Methanosarcina* were enriched after 40 days of acetate amendment. The increased abundance of *Methanosarcina* corresponded with an accumulation of methane in the groundwater. In order to determine whether *Methanosarcina* species could be participating in U(VI) reduction in the subsurface, cell suspensions of *Methanosarcina barkeri* were incubated in the presence of U(VI) with acetate provided as the electron donor. U(VI) was reduced by metabolically active *M*. *barkeri* cells; however, no U(VI) reduction was observed in inactive controls. These results demonstrate that *Methanosarcina* species could play an important role in the long-term bioremediation of uranium-contaminated aquifers after depletion of Fe(III) oxides limits the growth of *Geobacter* species. The results also suggest that *Methanosarcina* have the potential to influence uranium geochemistry in a diversity of anaerobic sedimentary environments.

**Electronic supplementary material:**

The online version of this article (10.1007/s00248-018-1165-5) contains supplementary material, which is available to authorized users.

## Introduction

Injection of acetate into the groundwater of uranium-contaminated aquifers has been shown to be an effective way to stimulate microbially mediated reductive precipitation of soluble U(VI) to poorly soluble U(IV) [1–3]. A wide diversity of microorganisms are capable of U(VI) reduction [4–9] but only *Geobacter* species have been shown to reduce U(VI) with acetate as an electron donor. Although growth with acetate as the electron donor and U(VI) as the electron acceptor is possible [4], the low concentrations of U(VI), even in heavily contaminated subsurface environments requires that microbes use other forms of respiration as their primary means of energy conservation [10]. *Geobacter* species grow rapidly in the initial phases of subsurface uranium bioremediation with added acetate because Fe(III) oxides are typically abundant in subsurface environments [1, 11–14] and *Geobacter* species outcompete other Fe(III) reducers under conditions of high acetate availability [15, 16]. However, the potential for other microorganisms to contribute to acetate oxidation coupled to U(VI) reduction, especially after the Fe(III) oxides that support *Geobacter* growth are depleted, has not been intensively investigated. Sulfate reducers that can reduce U(VI) have been identified, but none of these are known to use acetate as an electron donor [5, 7, 9, 17]. Furthermore, relying on sulfate reducers to reduce U(VI) may not be a good long-term strategy because acetate additions can rapidly deplete sulfate from groundwater [18–20].

Unlike Fe(III)- and sulfate-reducers, methanogens can thrive for long periods of time in organic-rich environments without external inputs of electron acceptors because they can conserve energy either from acetate dismutation or from the reduction of carbon dioxide, an electron acceptor generated by fermentation in their environment. If methanogens were capable of U(VI) reduction then this would make long-term in situ bioremediation of U(VI) a more attractive practice. To our knowledge, U(VI) reduction by methanogens has not been previously described. Previous studies have shown that methanogens can transfer electrons to various Fe (III) forms [21–26], as well as vanadate [27], molecular sulfur [28] and quinones [22, 29]. However, acetate has not been shown to serve as an electron donor for these processes.

Evidence for methane production in response to acetate amendments during in situ uranium bioremediation [30] led us to investigate the potential for methanogens to further contribute to uranium bioremediation. The results suggest that *Methanosarcina* species that can couple the oxidation of acetate to the reduction of U(VI) might aid in the bioremediation process.

## Materials and Methods

### Description of Sampling Site

The Rifle 24-acre experimental site is located close to the Colorado River, on the premises of an earlier uranium ore processing facility. Uranium concentrations in the water table of the Rifle aquifer are 2–8 times higher than the drinking water contamination limit (0.126 μM) established by the uranium mill tailings remedial action (UMTRA). A detailed review of geochemical characteristics of the site has already been published [31] and in situ bioremediation of U(VI) has been intensely studied at this site [1–3]. Similar to previous years, acetate was injected into the subsurface at a concentration of ~ 15 mM between August and October, 2011 and monitored from six different wells [32]. Groundwater and sediments for this study were collected from well CD-01 (a down gradient well) and a background well (CU-01) that never received any acetate additions.

### Nucleic Acid Extraction and cDNA Preparation

For nucleic acid extraction, it was first necessary to concentrate 50 L of groundwater by impact filtration on 293 mm diameter Supor membrane disc filters with pore sizes of 1.2 and 0.2 μm (Pall Life Sciences). All filters were placed into whirl-pack bags, flash frozen in a dry ice/ethanol bath, and shipped on dry ice back to the laboratory where they were stored at – 80 °C. RNA was extracted from the filters using a modified phenol–chloroform method, as previously described [12]. DNA was extracted from the filters with the FastDNA SPIN Kit for Soil (MP Biomedicals, Santa Ana, CA) according to the manufacturer’s instructions.

Extracted RNA and DNA were quantified with a NanoDrop spectrophotometer (Thermo Scientific, Wilmington, DE, USA) and stored at – 80 °C until further analyses. A DuraScript enhanced avian RT single-strand synthesis kit (Sigma, Sigma-Aldrich, St Louis, MO, USA) was used to generate cDNA from RNA, as previously described [32]. In order to ensure that RNA samples were not contaminated with DNA, PCR with primers targeting the 16S rRNA gene was conducted on RNA samples that had not undergone reverse transcription.

### PCR Amplification Parameters and Microbial Community Analysis

For clone library construction, fragments from the *mcrA* gene which codes for the large subunit of methyl CoM reductase and the 16S rRNA gene were amplified from cDNA with mcrAf/mcrAr primers [33] and with 344f/915r [34] (ESM 1: Supplementary Table S1). Amplicons were ligated into the pCR-TOPO2.1 TA cloning vector according to the manufacturer’s instructions (Invitrogen, the Netherlands). Inserts from the recombinant clones were directly amplified by PCR with M13 primers, purified and sequenced at the University of Massachusetts sequencing facility.

### Quantification of *MethanosarcinamcrA *Transcript Abundance

The quantitative PCR primer set (msa_mcrA173f/271r) targeted *mcrA* genes from *Methanosarcina* species found in the Rifle subsurface and was designed according to the manufacturer’s specifications (Applied Biosystems) (ESM 1: Supplementary Table S1). Quantitative PCR amplification and detection was performed with the 7500 Real Time System (Applied Biosystems) using cDNA made by reverse transcription from total RNA extracted from groundwater collected during the bioremediation experiment. Each reaction mixture consisted of a total volume of 25 μl and contained 1.5 μl of the appropriate primers (stock concentration 1.5 μM), 5 ng cDNA, and 12.5 μl Power SYBR Green PCR Master Mix (Applied Biosystems). All qPCR experiments followed MIQE guidelines [35] and qPCR efficiencies were 98%. Optimal thermal cycling parameters consisted of an activation step at 50 °C for 2 min, an initial 10 min denaturation step at 95 °C, and 50 cycles of 95 °C for 15 s and 60 °C for 1 min. A dissociation curve generated by increasing the temperature from 58 to 95 °C at a ramp rate of 2% showed that the PCR amplification process yielded a single predominant peak, further supporting the specificity of the qPCR primer pair.

### Phylogenetic Analysis

*mcrA* gene sequences were compared to Genbank nucleotide and protein databases with the BLASTn and BLASTx algorithms [36, 37]. Alignments were generated with MAFFT [38] and PRANK [39] algorithms. The phylogenetic tree was inferred with the Maximum Likelihood method using MEGA7 software [40]. The percentage of replicate trees in which the associated taxa clustered together in the bootstrap test (100 replicates) is shown next to the branches [41]. All positions with less than 95% coverage were eliminated and a total of 117 positions were considered in the final dataset.

Nucleotide sequences of *mcrA* genes used for phylogenetic analyses have been deposited in the Genbank database under accession numbers MF616623-MF616647.

### U(VI) Reduction Studies

*Methanosarcina barkeri* (DSM 800) was selected for U(VI) reduction studies because sequences most similar to this strain were significant members of the *Methanosarcina* community (37% of the sequences). Although *M*. *barkeri* was isolated from an anaerobic sewage digester [42], it grows in freshwater medium and can utilize acetate as a substrate for methanogenesis, similar to methanogens likely to be enriched from the acetate-amended Rifle aquifer. In addition, the majority of studies examining reduction of extracellular electron acceptors by *Methanosarcina* have focused on *M*. *barkeri* [22–25].

Batch cultures of 500-mL *M*. *barkeri* were grown under strictly anaerobic conditions [42] on modified DSMZ medium 120 [43] with acetate (40 mM) as substrate, and incubated at 37 °C for ~ 3 weeks. Cultures were harvested when they reached an optical density at 600 nm of 0.19. All cell suspension preparations were performed in an anaerobic chamber to minimize oxygen exposure. Cells were pelleted by centrifugation for 10 min at 4000 × g in a Sorval RC 5B Plus centrifuge. These pellets were then washed twice in anoxic phosphate depleted buffer (PDB), which consisted of the following salts 0.2 g/L MgSO_4_ × 7H_2_O, 0.025 g/L CaCl_2_ × 2H_2_O, 1 g/L NaCl, and 2 g/L NaHCO_3_. Cell pellets were then resuspended in 10-mL anoxic PDB to a cell density of ~ 0.4–0.5 at 600 nm. To generate heat-killed cells, 3 mL of this suspension was autoclaved at 122 °C for 30 min. Six replicates were prepared by diluting 1 mL of the cell-suspension in 9-mL PDB buffer. For the heat-killed incubation, 1-mL autoclaved cell suspension was diluted in 9-mL PDB buffer. Sulfide (0.5 mM) was added to all inoculated tubes to ensure anoxic conditions. Acetate (40 mM) was also added to the tubes to fuel methanogenic activity. Triplicate live cell suspensions (active cells) and triplicate heat-killed controls (heat-killed cells) were incubated at 37 °C. The other three live cell suspensions were incubated at 4 °C (inactive cells). All cell suspensions were incubated with 0.2 mM U^6+^ prepared from a stock of uranyl-acetate (5 mM). Cell densities were determined with a bench top spectrophotometer, by absorbance measurements at 600 nm with mili-Q water as a blank.

The ability of *Methanosarcina barkeri* to reduce U(VI) was verified with U(VI) depletion measurements carried out on different cell suspensions over the course of 24 h. Samples (0.1 mL) were retrieved anaerobically and diluted in 14.9 mL anoxic bicarbonate (100 mM) and 14.9 mL Uraplex solution. Concentrations of U(VI) were then measured with a kinetic phosphorescence analyzer, as previously described [44].

### Chemical Analyses

Groundwater samples for geochemical analyses were collected after purging 12 L of groundwater from the wells with a peristaltic pump. The phenanthroline method [45] was used to determine ferrous iron concentrations. Sulfate and thiosulfate concentrations were measured with an ion chromatograph (ICS-2100, Dionex, CA) equipped with an AS18 column under isocratic elution with 32 mM KOH as the eluent. Acetate concentrations were determined with a high performance liquid chromatograph equipped with an ion exclusion HPX-87H column (Biorad, Hercules, CA) using 8 mM sulfuric acid as eluent. In situ methane production was monitored as previously described [30]. Methane in the headspace of sediment/groundwater incubations was measured as previously described [43] using a gas chromatograph with a flame ionization detector (Shimadzu, GC-8A).

## Results and Discussion

### Evidence for Acetoclastic Methanogenic Activity During Acetate Amendments

**Fig. 1:**
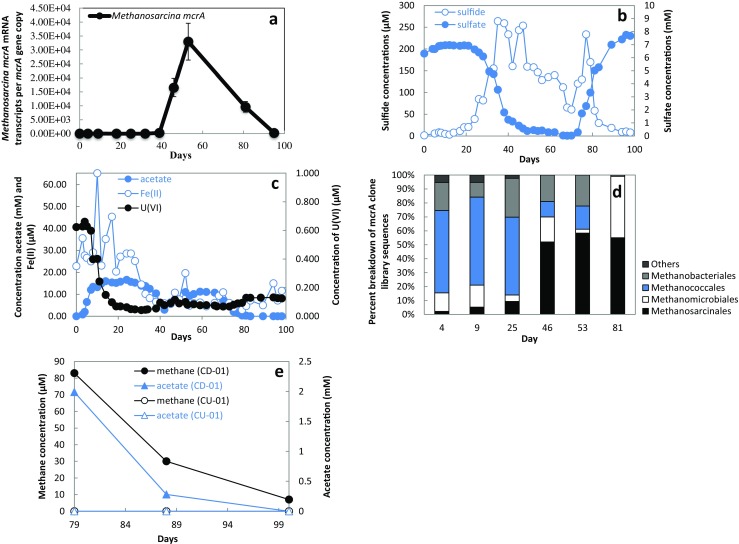
The injection of acetate into a uranium-contaminated aquifer, triggered acetate utilization coupled with iron reduction, sulfate reduction, and methanogenesis. **a** Quantitative RT-PCR of *Methanosarcina mcrA* mRNA transcripts normalized against *Methanosarcina mcrA* gene copy numbers recovered in the groundwater over the course of 100 days. **b** Concentrations of hydrogen sulfide (μM) and sulfate (mM) detected in the groundwater. **c** Concentrations of acetate (mM), Fe(II) (μM), and U(VI) (μM) detected in the groundwater. **d** Proportion of *mcrA* sequences from various methanogenic families found in cDNA clone libraries assembled from RNA extracted from groundwater at different points during the experiment. **e** Concentrations of methane and acetate in an active well (CD-01) and a background well (CU-01) on days 79, 89, and 100. For further reference to geochemical parameters and cDNA clone libraries, see Holmes et al. 2014

Methanogens that utilize acetate are restricted to the order Methanosarcinales [46]. In order to determine whether the addition of acetate could promote the growth of acetoclastic methanogens in a uranium-contaminated aquifer, the activity of Methanosarcinales was investigated by monitoring *Methanosarcina mcrA* gene transcript abundance. Before day 39, fewer than two *Methanosarcina mcrA* mRNA transcripts were detected per *mcrA* gene copy number in the groundwater (Fig. 1a). However, by day 46, *Methanosarcina mcrA* transcripts increased by four orders of magnitude to 1.7 × 10^4^ transcripts per gene copy. This increase in *Methanosarcina* coincided with a steep decline in groundwater sulfate concentrations (Fig. 1b). Although sulfate reducers and methanogens compete for acetate [47, 48], high concentrations of acetate in the groundwater (Fig. 1c) made it unlikely that growth of Methanosarcinales in the subsurface was being restricted by competition for acetate.

The increase in Methanosarcinales coincided with an increase in free sulfide in the groundwater, producing highly reducing conditions that favor the growth of methanogens*.* Another consideration is the slow growth rate of Methanosarcinales, which might have limited their growth after acetate injections even under the most favorable conditions. The lack of sufficient reducing conditions coupled with the slow growth rate of Methanosarcinales may explain the finding that although acetate concentrations were high during the Fe(III) reducing phase of the experiment (days 0–33) (Fig. 1c), the number of Methanosarcinales sequences stayed low until sulfate reduction became the primary subsurface metabolism (Fig. 1a, d). The increase in abundance of Methanosarcinales was later followed by a decline, which coincided with acetate limitation associated with the halt in acetate injections on day 68.

Measurements of methane concentrations in the groundwater were not initiated until day 79 (Fig. 1e). The high concentration of methane at this time demonstrated that methanogens had been active in the preceding days. Methane concentrations steeply declined over time coincident with the steep decline in acetate availability.

### Phylogenetic Analysis of the In Situ *Methanosarcina* Community

**Fig. 2:**
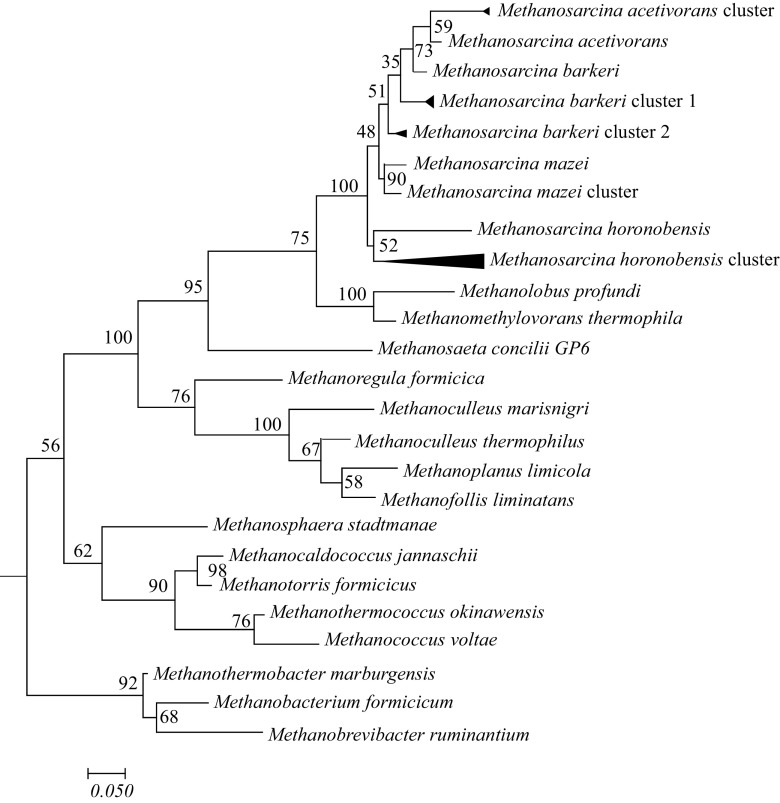
Phylogenetic tree generated with the maximum likelihood algorithm comparing translated *mcrA* mRNA transcript sequences to McrA protein sequences from known methanogenic archaea. Bootstrap values were generated with 100 replicates and *Methanobacterium formicum*, *Methanothermobacter marburgensis*, and *Methanobrevibacter ruminantium* were used as outgroups

Methanosarcinales accounted for the majority of methanogenic *mcrA* transcripts recovered from the groundwater on days 46 through 81 (Fig. 1d). The most abundant *Methanosarcina mcrA* cDNA sequences recovered from groundwater during this period clustered with *M*. *horonobensis* (48.2%) and *M*. *barkeri* (37% of the sequences) (Fig. 2). Other *Methanosarcina mcrA* cDNA sequences detected included sequences most similar to *M*. *mazei* (11.1% of the sequences), and *M*. *acetivorans* (3.7% of the sequences). More than half of these sequences clustered with acetoclastic *Methanosarcina* that are unable to use formate or hydrogen as substrates for growth (i.e., *M*. *horonobensis* and *M*. *acetivorans*) [49, 50] suggesting that they might be growing during the in situ U(VI) experiment via acetate dismutation.

### U(VI) Reduction by Metabolically Active *Methanosarcina* Cells

**Fig. 3:**
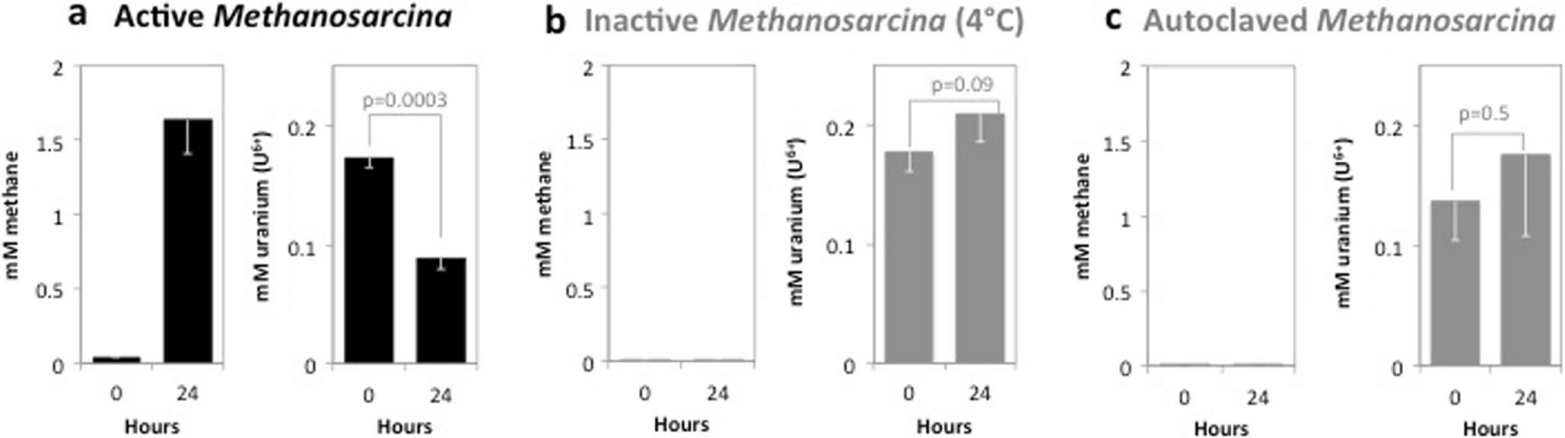
Uranium U(VI) reduction by metabolically active *Methanosarcina* cells. Metabolically active cells which were defined as such because they were producing methane from acetate were able to convert 51% of U(VI) to U(IV) (**a**) whereas metabolically inactive cells kept at 4 °C in the same medium did not produce methane and also did not convert U(VI) to U(IV) (**b**), and neither did autoclaved cell suspensions from the same culture (**c**). The difference between original concentrations of U(VI) and the amount recovered in metabolically active cell suspensions after 24 h of exposure was statistically different (*p* = 0.0003)

To evaluate whether *Methanosarcina* species might be capable of U(VI) reduction, cell suspensions of *M*. *barkeri* were incubated with acetate as the electron donor and 200-μM U(VI) as a potential electron acceptor. Within 1 day, the cells produced 1.6-mM methane while depleting 51% of the provided U(VI) (Fig. 3a). In contrast, cell suspensions incubated at 4 °C or autoclaved prior to incubation, did not produce methane or remove U(VI) (Fig. 3b, c). These results indicated that U(VI) removal could be attributed to U(VI) reduction by metabolically active cells.

### Implications

Our findings that acetate additions during in situ uranium bioremediation promotes the growth of *Methanosarcina* and that a *Methanosarcina* can reduce U(VI) has important implications for the design of long-term in situ uranium bioremediation strategies. Previous interpretations of U(VI) reduction during acetate-amendment at the Rifle, Colorado study site have focused on the U(VI) reduction capacity of *Geobacter* species because of their prevalence at the site [1–3, 51–54] and because the sulfate-reducers that are enriched with acetate amendments [18, 19, 55, 56] are not likely to be effective U(VI) reducers. In fact, there has yet to be a description of an acetate-utilizing sulfate-reducing microorganism capable of U(VI) reduction. The results presented here suggest that *Methanosarcina* may also contribute to U(VI) reduction in the field experiments. Unlike *Geobacter* species, *Methanosarcina* do not require an external electron acceptor for acetate metabolism. Therefore, in long-term in situ uranium bioremediation, *Methanosarcina* may emerge as an important microbial catalyst for uranium removal.

Furthermore, microbial reduction of U(VI) may play an important role in the uranium geochemistry of a diversity of sedimentary environments [4]. Thus, the potential contribution of *Methanosarcina* to U(VI) reduction in anaerobic environments should be considered.

## Electronic supplementary material

ESM 1 (DOCX 25 kb)
