# Supplementary material for: Potential for Methanosarcina to Contribute to Uranium Reduction during Acetate-Promoted Groundwater Bioremediation
Source: Microb Ecol. 2018 Mar 2;76(3):660–7. doi: 10.1007/s00248-018-1165-5 (PMC6132540; doi:10.1007/s00248-018-1165-5)
Supplement: Supplementary file 1 — (DOCX 25 kb) [file 248_2018_1165_MOESM1_ESM.docx]

Supplemetary Table S1. Primers used in this study

| Primer name | Primer sequence | Target | References |
| --- | --- | --- | --- |
| mcrA_F | GTGGTGTMGGATTCACACARTAYGCWACAGC | mcrA Euryarchaea | (Luton et al., 2002) |
| mcrA_R | TTCATTGCRTAGTTWGGRTAGTT | mcrA Euryarchaea | (Luton et al., 2002) |
| 344f* | ACGGGGYGCAGCAGGCGCGA | SSU Euryarchaea | (Casamayor et al., 2002) |
| 915r* | GTGCTCCCCCGCCAATTCCT | SSU Euryarchaea | (Casamayor et al., 2002) |
| msa_mcrA173f | ACCTTTCGGACATCATCCAG | mcrA Rifle Methanosarcina | This study |
| msa_mcrA271r | TCTGGTCGTAAAGCATGCAG | mcrA Rifle Methanosarcina | This study |
| M13F | GTAAAACGACGGCCAG | Plasmid | Addgene (http://www.addgene.org/) |
| M13R | CAGGAAACAGCTATGAC | Plasmid | Addgene (http://www.addgene.org/) |

*Primer pair 344f/915r matches 78.4% Euryarchaea sequences in the Silva SSU database of which 82% Methanobacteria, 86% Methanococci, 78% Methanomicrobia (72% Methanocellales, 80% Methanomicrobiales, 83% Methanosarcinales), 100% Methanopyri.
